# The large carpenter bees of central Saudi Arabia, with notes on the biology of *Xylocopa sulcatipes* Maa (Hymenoptera, Apidae, Xylocopinae)

**DOI:** 10.3897/zookeys.201.3246

**Published:** 2012-06-14

**Authors:** Mohammed A. Hannan, Abdulaziz S. Alqarni, Ayman A. Owayss, Michael S. Engel

**Affiliations:** 1 Department of Plant Protection, College of Food and Agriculture Sciences, King Saud University, Riyadh 11451, PO Box 2460, KSA; 2 Division of Entomology, Natural History Museum, and Department of Ecology & Evolutionary Biology, 1501 Crestline Drive – Suite 140, University of Kansas, Lawrence, Kansas 66049-2811, USA

**Keywords:** Apoidea, Anthophila, Xylocopini, Arabian Peninsula, systematics, biology, host plants, nesting

## Abstract

The large carpenter bees (Xylocopinae, *Xylocopa* Latreille) occurring in central Saudi Arabia are reviewed. Two species are recognized in the fauna, *Xylocopa (Koptortosoma) aestuans* (Linnaeus) and *Xylocopa (Ctenoxylocopa) sulcatipes* Maa. Diagnoses for and keys to the species of these prominent components of the central Saudi Arabian bee fauna are provided to aid their identification by pollination researchers active in the region. Females and males of both species are figured and biological notes provided for *Xylocopa sulcatipes*. Notes on the nesting biology and ecology of *Xylocopa sulcatipes* are appended. As in studies for this species from elsewhere, nests were found in dried stems of *Calotropis procera* (Aiton) (Asclepiadaceae) and *Phoenix dactylifera* L. (Arecaceae).

## Introduction

The tribe Xylocopini comprises the large carpenter bees (Xylocopinae: *Xylocopa* Latreille) species of which principally nest in dead wood (including the wood of human constructions), bamboo culms, and other similar substrates (e.g., Hurd 1958, 1978; Hurd and Moure 1960, 1963; Sakagami and Laroca 1971; Gerling et al. 1983, 1989; Maeta et al. 1985, 1996; Raju and Rao 2006; Boontop et al. 2008; Gonzalez et al. 2009; Pereira and Garófalo 2010; Prager and Hunter 2011), and Le Goff (2004) even records a species emerging from the wood of a Senegalese ceremonial mask. Owing to their conspicuousness, interesting biology and behavior, and agricultural potential (e.g., Keasar 2010), the group has received increased systematic scrutiny, although most recent work has focused on relationships among the tribes of the subfamily or those of the constituent genera and subgenera (e.g., Minckley 1998; Engel 2001, in press; Leys et al. 2002; Flores-Prado et al. 2010). Meanwhile, the number of detailed, species-level revisions has lagged over the last 15 years (although when done, there are some very nice examples: e.g., Leys 2000) despite the fact that it is at this level for which the documentation and interpretation of biological phenomena is most critical (e.g., Engel 2011). Today, the diversity of carpenter bees in many areas of the world remains largely unexplored.

As part of an on-going effort to survey the bee fauna and pollinator resources of the Kingdom of Saudi Arabia and to eliminate the taxonomic impediment for working on this diverse region, we have begun with surveys of the melittofauna from central Saudi Arabia. Herein we provide a brief contribution to this larger effort by documenting the species of the large carpenter bees occurring in this area and as an aid to studies of wild bee pollination already underway (Hannan et al. in prep.). Two species are recognized from the region, *Xylocopa (Koptortosoma) aestuans* (Linnaeus) and *Xylocopa (Ctenoxylocopa) sulcatipes* Maa, although the latter may be frequently found misidentified in some collections as *Xylocopa (Xylomelissa) hottentotta* Smith or *XC*.) *fenestrata* (Fabricius) (e.g., Al-Ahmadi and Salem 1999). *Xylocopa hottentotta* is an entirely unrelated species of African distribution (Eardley 1983, 1993), while *Xylocopa fenestrata* is certainly very closely allied to *Xylocopa sulcatipes* but occurs more easterly and southerly (Maa 1970). The latter species is quite similar but can be readily distinguished on the basis of the male terminalia (Maa 1970, and figures herein, *vide infra*). In total five nominal species have been recorded from throughout the Arabian Peninsula [e.g., Al-Ahmadi and Salem 1999: recorded as *Xylocopa aestuans*, *Xylocopa hottentotta*, *Xylocopa caffra* (Linnaeus), and *Xylocopa valga*?], but many of these seem to be misidentifications (e.g., *Xylocopa hottentotta*) and thorough collecting and new identifications, particularly with comparisons to holotypes, is desperately needed. Diagnoses, figures (particularly the male terminalia), and keys are provided so as to aid regional entomologists in the identification of their material. In addition, we append observations on the biology and ecology of *Xylocopa sulcatipes*.

## Material and methods

Material examined herein is deposited in the King Saud University Museum of Arthropods, Plant Protection Department, College of Food and Agriculture Sciences, King Saud University, Riyadh, Kingdom of Saudi Arabia (KSMA) and Division of Entomology (Snow Entomological Collections), University of Kansas Natural History Museum, Lawrence, Kansas, USA (SEMC). Photomicrographs were prepared using a Nikon D1x digital camera attached to an Infinity K-2 long-distance microscope lens. Morphological terminology in the diagnoses follows that of Engel (2001) and Michener (2007). Herein we follow the supraspecific classification of Xylocopini advocated by Minckley (1998) and Michener (2007).

The nesting biology of *Xylocopa sulcatipes* was studied in Amariah, approximately 25 km northwest of Riyadh, from September 2010 through December 2011. Nests were found on 6 June 2011 at the base of a large hill near an agricultural farm near Wadi Amariah and the highway to Riyadh. Prior to collection the nests were observed for at least an hour to note the coming and going of bees. Most nests were located around 9:00am and collected around 12:00pm. Nests were sealed with plastic and brought to the lab for dissection and study. During four visits (6, 12, 19 June and 28 September 2011) a total of 13 nests were collected (Table 1). Nests were in the dead branches of local milkweeds [*Calotropis procera* (Aiton) (Asclepiadaceae), more widely known as the “Apple of Sodom”] growing in a sparsely vegetative desert area and among date palms, *Phoenix dactylifera* L. (Arecaceae). Nests were measured, sketched, and photographed, and the inhabitants deposited in the KSMA repository.

**Table 1.** Measurements of sampled nests of *Xylocopa (Ctenoxylocopa) sulcatipes* Maa from central Saudi Arabia collected in dead wood of two plants. Means are given with standard deviations. n = number of nests sampled for each metric.

	Asclepiadaceae	Arecaceae
Metric	*Calotropis procera* (Aiton)	*Phoenix dactylifera* L.
Branch length (cm)	147.75±74.59 (n=8)	57.33±6.53 (n=6)
Nest entrance (mm)	9.06±0.78 X 8.85±0.86 (n=8)	10.17±2.17 X 11±2 (n=7)
Height of nest from ground (cm)	83.50±30.3 (n=8)	400±0 (n=6)
Length of nest (cm)	23.61±14.93 (n=7)	11.05±4.36 (n=6)
Branch diameter at nest (cm)	1.88±0.38 (n=8)	6.83±0.41 (n=6)
Internal diameter of nest (cm)	1.28±0.19 (n=8)	1.77±0.12 (n=6)
Number of cells/nest	6.60±5.6 (n=5)	4.50±1.64 (n=6)
Length of cells (mm)	18.8±1.63 (n=30)	20.11±1.37 (n=19)

## Systematics

### Genus *Xylocopa* Latreille

Subgenus *Koptortosoma* Gribodo

This is the largest and most widespread subgenus of carpenter bees, with at least 196 recognized species ranging throughout Subsaharan Africa to the Mediterranean countries of that continent, Dalmatia, the Arabian Peninsula, southwestern Asia, and southern Asia east to the Philippines, Taiwan, and Japan, and south through Indonesia, New Guinea, and the Bismarck Archipelago to southernmost Australia (Michener 2007). The subgenus can be recognized by the female mesoscutellum having a sharp truncation overhanging the metanotum (as in subgenus *Mesotrichia* Westwood) and surpassing the posterior margin of the latter, and males with unmodified tegulae (elongate in *Mesotrichia*) (Michener 2007).

#### Xylocopa (Koptortosoma) aestuans

(Linnaeus)

http://species-id.net/wiki/Xylocopa_aestuans

Figs 1 [Fig F2] -11

Apis aestuans Linnaeus, 1758: 579 [♀].
Xylocopa aestuans (Linnaeus); Illiger 1806: 151.

##### Diagnosis.

**Figures 1–4.**
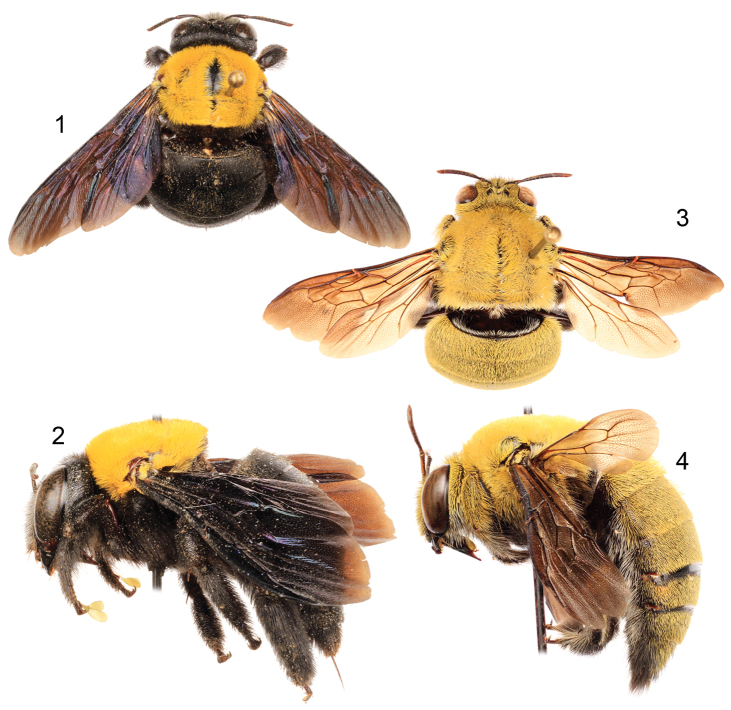
Habitus photomicrographs of *Xylocopa (Koptortosoma) aestuans* (Linnaeus) from central Saudi Arabia. **1** Female, dorsal **2** Female, lateral **3** Male, dorsal **4** Male, lateral.

**Figures 5–6. F2:**
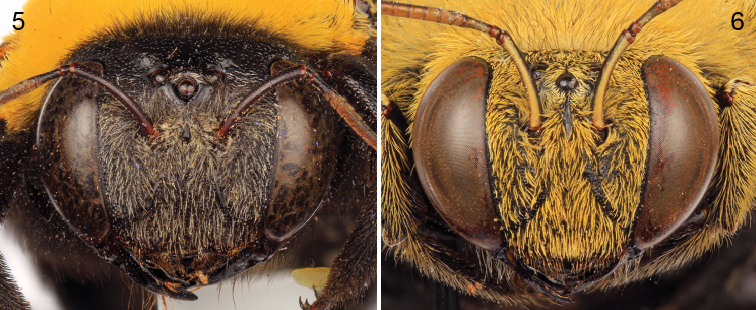
Faces of *Xylocopa (Koptortosoma) aestuans* (Linnaeus) from central Saudi Arabia. **5** Female **6** Male.

**Figures 7–11.**
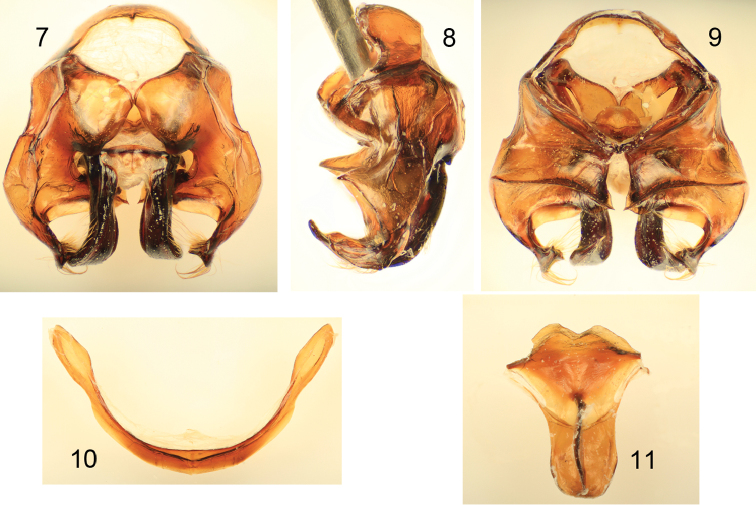
Male terminalia of *Xylocopa (Koptortosoma) aestuans* (Linnaeus) from central Saudi Arabia. **7** Genital capsule, dorsal aspect **8** Genital capsule, lateral aspect **9** Genital capsule, ventral aspect **10** Seventh metasomal sternum **11** Eighth metasomal sternum.

*Xylocopa aestuans* can be most readily distinguished from other Saudi Arabian large carpenter bees by the following: female face with largely white or pale pubescence (Fig. 5), mesosomal dorsum densely covered by yellow pubescence obscuring underlying integument (Figs 1, 2); mandible bidentate at apex; posterodorsal margin of mesoscutellum projecting beyond posterior margin of metanotum; pygidial plate unarmed. Male covered by dense yellow pubescence (Figs 3, 4, 6); first metasomal tergum with subhorizontal dorsal surface abruptly and angulately separated from declivitous anterior surface; gradulus of first metasomal tergum transverse, lateral extremities not directed posteriorly; male terminalia as in figures 7–11.

##### Comments.

*Xylocopa aestuans* is one of the widespread and ubiquitous of large carpenter bee species. There has been considerable debate regarding the identity of the species of *Koptortosoma* similar to *Xylocopa aestuans* (i.e., considering them synonyms, subspecies, or separate species), with different authors of varying opinions how to segregate the minor variation into natural taxonomic entities (e.g., Lieftinck 1964). The Saudi Arabian populations have been at times considered to the belong to the largely African, *Xylocopa pubescens* Spinola, although the genitalia of those populations are quite dissimilar from true *Xylocopa pubescens*. Indeed, the genitalia (Figs. 7–11) and other characters are certainly more alike the more easterly populations of *Xylocopa aestuans* and there seems little reason at this time to not consider the central Saudi Arabian populations as such, as was done by Shalaby (1961). The species has also been recorded from the United Arab Emirates (Harten 2005; Dathe 2009). Biological accounts, largely from India or Southeast Asia, have been provided by Dover (1924), Monod (1977), Binti (1992), El-Borollosy and Ismail (1972: note that these observations may be of *Xylocopa pubescens*, the identity of their material requires checking), and Punekar et al. (2010).

### Subgenus *Ctenoxylocopa* Michener

This is a widespread, albeit not very diverse, subgenus of Old World carpenter bees (Maa 1970; Michener 2007). The lineage can be recognized by the prolonged posterior pronotal lobes and elevated process of the spiracles on the third metasomal tergum in males, while females are noteworthy for the combination of a row of tubercles along each margin of the metabasitibial plate, the tridentate mandibles, and a single spine on the outer apex of the metatibia.

#### Xylocopa (Ctenoxylocopa) sulcatipes

Maa

http://species-id.net/wiki/Xylocopa_sulcatipes

**Figures 12–15.**
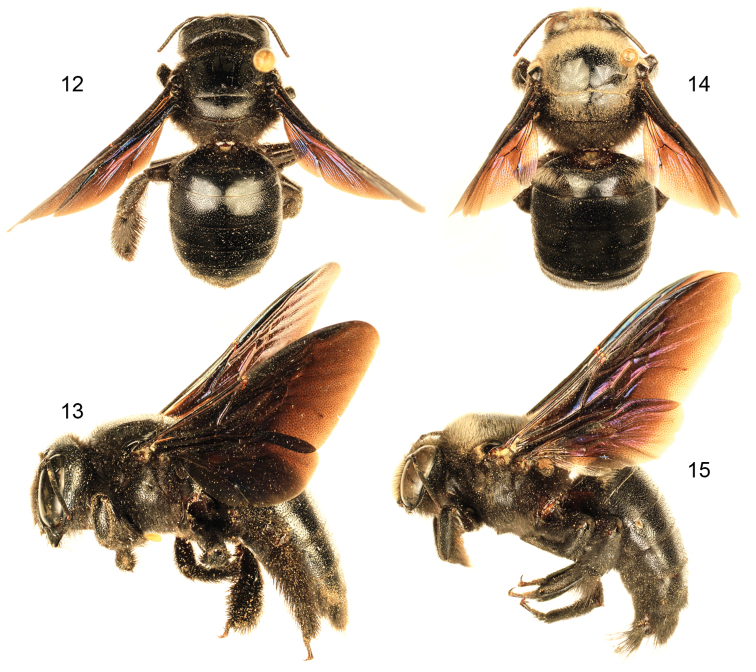
Habitus photomicrographs of *Xylocopa (Ctenoxylocopa) sulcatipes* Maa from central Saudi Arabia. **12** Female, dorsal **13** Female, lateral **14** Male, dorsal **15** Male, lateral.

Figs 12 [Fig F5] -22

Xylocopa (Ctenoxylocopa) sulcatipes Maa, 1970: 739 [♂♀].

##### Diagnosis.

**Figures 16–17. F5:**
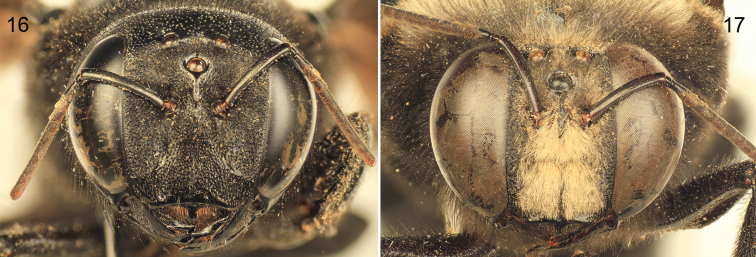
Faces of *Xylocopa (Ctenoxylocopa) sulcatipes* Maa from central Saudi Arabia. **16** Female **17** Male.

**Figures 18–22.**
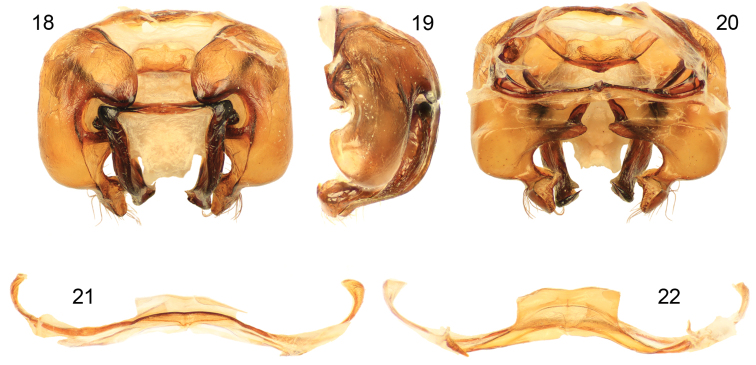
Male terminalia of *Xylocopa (Ctenoxylocopa) sulcatipes* Maa from central Saudi Arabia. **18** Genital capsule, dorsal aspect **19** Genital capsule, lateral aspect **20** Genital capsule, ventral aspect **21**  Seventh metasomal sternum **22** Eighth metasomal sternum.

*Xylocopa sulcatipes* can be most readily distinguished from other Arabian large carpenter bees by the following: Female with face with largely black pubescence (Fig. 16), mesosomal dorsum largely covered by black pubescence not obscuring underlying integument (Figs 12, 13); mandible tridentate at apex; mesoscutellum not projecting over metanotum, apical margin rounded in profile; pygidial plate armed on each side with subapical spine. Male covered by largely fuscous to black pubescence except face, dorsum of mesosoma, and apicolateral patches of first metasomal tergum with predominantly white or pale setae (Figs 14, 15, 17); first metasomal tergum with subhorizontal dorsal surface rounding into declivitous anterior surface; gradulus of first metasomal tergum laterally curved posteriorly; male terminalia as in figures 18–22.

##### Comments.

Maa (1970) recorded *Xylocopa sulcatipes* from Saudi Arabia, Yemen, Israel, and Transcaspia (likely northern Iran, or southwesternmost Turkmenistan), while Vicidomini (2004) gave localities in Jordan. The records of *Xylocopa fenestrata* from the United Arab Emirates (Dathe 2009) are likely *Xylocopa sulcatipes*, and this material should be dissected and compared with the images herein (Figs. 18–22) as well as those of Maa (1970). We have found that Maa’s (1970) characterization of the terminalic differences holds well for observed populations and the species he recognized appear to be good (Engel pers. obs.).

### Key to the Species of *Xylocopa* in Central Saudi Arabia

**Table:** 

1	Males	2
–	Females	3
2	Bee covered by dense yellow pubescence; first metasomal tergum with subhorizontal dorsal surface abruptly and angulately separated from declivitous anterior surface; gradulus of first metasomal tergum transverse, lateral extremities of gradulus not directed posteriorly; terminalia as in figures 7–11	*Xylocopa aestuans* (Linnaeus)
–	Bee covered by largely fuscous to black pubescence except face, dorsum of mesosoma, and apicolateral patches of first metasomal tergum with predominantly white or pale setae; first metasomal tergum with subhorizontal dorsal surface rounding into declivitous anterior surface; gradulus of first metasomal tergum laterally curved posteriorly; terminalia as in figures 18–22	*Xylocopa sulcatipes* Maa
3	Mesosomal dorsum densely covered by yellow pubescence obscuring underlying integument; face with largely white or pale pubescence; pygidial plate unarmed; posterodorsal margin of mesoscutellum projecting beyond posterior margin of metanotum; mandible bidentate at apex	*Xylocopa aestuans* (Linnaeus)
–	Mesosomal dorsum largely covered by black pubescence not obscuring underlying integument; face with largely black pubescence; pygidial plate armed on each side with subapical spine; mesoscutellum not projecting over metanotum, apical margin rounded in profile; mandible tridentate at apex	*Xylocopa sulcatipes* Maa

### Biological Notes on *Xylocopa sulcatipes* at Amariah

**Figures 23–26.**
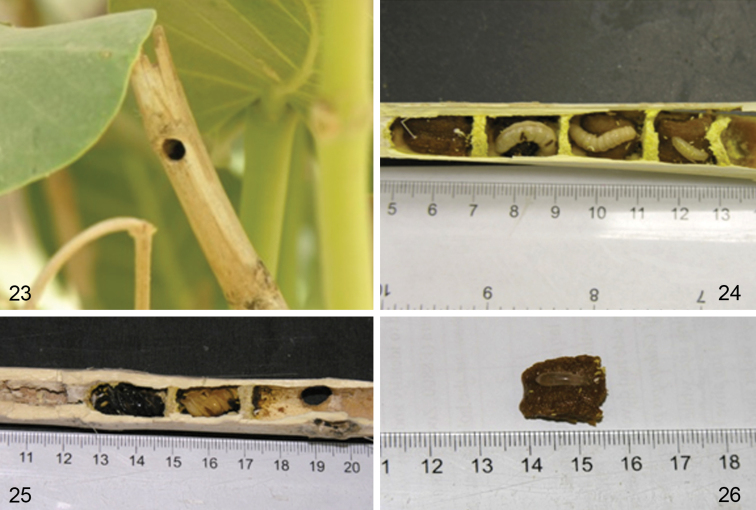
Photographs of nests of *Xylocopa (Ctenoxylocopa) sulcatipes* Maa in stems of *Calotropis procera* (Aiton) in central Saudi Arabia. **23** Nest entrance in stem of *Calotropis procera* in the wild **24** Opened nest with series of larvae in individual cells **25** Opened nest with pupae **26** Individual pollen mass with egg situated on top.

**Figure 27.**
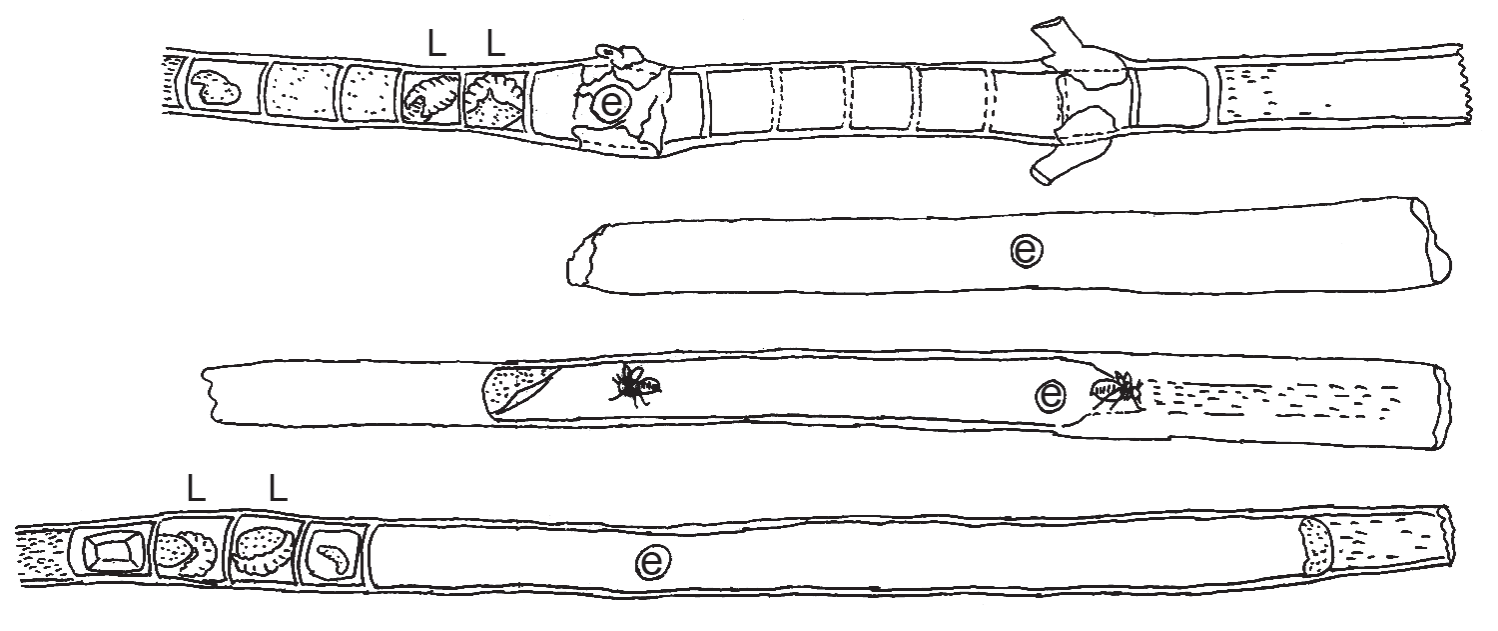
Diagrams of representative nests of *Xylocopa (Ctenoxylocopa) sulcatipes* Maa in stems of *Calotropis procera* (Aiton) in central Saudi Arabia. L = larva; e = nest entrance. Line illustrations by M.A. Hannan.

The biology of *Xylocopa sulcatipes* has been the focus of several extensive ecological and behavioral studies, principally in Israel (e.g., Eisikowitch 1986; Gerling et al. 1983, 1989; Hefetz 1983; Kronenberg and Hefetz 1984; Stark 1989, 1992a, b; Stark et al. 1990; Surholt et al. 1990; Velthuis 1987; Velthuis and Gerling 1980; Velthuis et al. 1984; Willmer 1988). Our observations do not differ from those of the previous studies except that we have focused more on the architecture of the nests rather than the particular ecology of the species, which is already well characterized. The species is bivoltine and foraged from March through November. The area around Amariah, where our observations were made, is a typical central Saudi Arabian desert environment. Vegetation is thinly scattered and comprised mostly native plants, including several promising foraging flowers and nesting sites throughout the season. Among these, *Calotropis procera* was found to be the most commonly used for nesting and provisioning resources. The pithy and rather straight stems of suitable diameter of *Calotropis procera* make them ideal for nest construction (e.g., Figs 23–27). Secondarily, *Phoenix dactylifera* was used as a nesting substrate (with five such nests collected). Detail measurements of the nests observed are provided in table 1. Given the different overall physical structure of these substrates it is not surprising that nests in *Calotropis procera* had a single, linear nest tube extending to each side of the entrance (Figs 23–27), while those in *Phoenix dactylifera* consisted of a more gallery-like structure, similar in this regard to the variation observed for *Xylocopa (Stenoxylocopa) artifex* Smith (Silveira 2002). In all of the nests the pollen masses were compact, well kneaded, and mixed with sufficient nectar to leave them moist (Fig. 26). Where observed, individual pollen loaves were fully consumed and the larvae defecated pellet-like feces which were placed to its back or at the bottom of the cell. As observed elsewhere for this species, cells were arranged linearly, but never with cells closer than 1–1.5 times an individual cell length from the nest entrance. Also similar to observations made elsewhere on this species (e.g., Gerling et al. 1989), some nests were found to comprise several newly emerged females along with an older female, all of whom participated in foraging but apparently built their own cells, although further observations are needed to clarify this point.

Although *Xylocopa sulcatipes*, like other *Xylocopa*, is polylectic, females were observed foraging mostly from *Calotropis procera* and it was there that males were seen to approach and grab females for mating. In addition to foraging at *Calotropis procera*, females were observed visiting *Reseda alba* L. (Resedaceae) and radish [*Raphanus sativus* L. (Brassicaceae)]. Given that species of *Xylocopa* may be useful for agricultural pollination (Keasar 2010) it may be beneficial for standing crops in central Saudi Arabia to be surrounded by suitable native vegetation including *Calotropis procera*, thereby providing ample nesting sites to encourage the establishment of sustainable and large populations of these bees. No associated organisms were found among the nests observed.

## Supplementary Material

XML Treatment for Xylocopa (Koptortosoma) aestuans

XML Treatment for Xylocopa (Ctenoxylocopa) sulcatipes
